# Alcohol policies, firearm policies, and suicide in the United States: a lagged cross-sectional study

**DOI:** 10.1186/s12889-021-10216-x

**Published:** 2021-03-01

**Authors:** Sharon M. Coleman, Marlene C. Lira, Jason Blanchette, Timothy C. Heeren, Timothy S. Naimi

**Affiliations:** 1 Biostatistics and Epidemiology Data Analytics Center (BEDAC), Boston University School of Public Health, 85 East Newton Street M921, Boston, MA USA; 2 Clinical Addiction Research and Education (CARE) Unit, Section of General Internal Medicine, Department ofaaaaaa Medicine, Boston Medical Center, 801 Massachusetts Ave., Second Floor, Boston, MA 02118 USA; 3 Department of Health Law, Policy & Management, Boston University School of Public Health, 715 Albany Street, Talbot Building T2W, Boston, MA USA; 4 Department of Biostatistics, Boston University School of Public Health, 801 Massachusetts Ave., Third Floor, Boston, MA USA; 5 Department of Community Health Sciences, Boston University School of Public Health, 801 Massachusetts Ave., Fourth Floor, Boston, MA USA

**Keywords:** Alcohol policies, Firearm policies, Suicide

## Abstract

**Background:**

Alcohol and firearms are commonly involved in suicide in the United States. State alcohol and firearm policies may impact alcohol and firearm related suicide, yet little is known about these relationships. This study examines relationships between state alcohol and firearm policies and suicides involving alcohol, guns, or both, and explores interactive policy associations.

**Methods:**

Alcohol policies were assessed with the Alcohol Policy Scale. Firearm policies were assessed using the Gun Law Scorecard from Giffords Law Center. Suicide data from the National Violent Death Reporting System in 2015 covered 22 states. State- and individual-level GEE Poisson and logistic regression models assessed relationships between policies and firearm- and/or alcohol-involved suicides with a 1-year lag.

**Results:**

In 2015, there were 8996 suicide deaths with blood alcohol concentration test results in the 22 included states. Of those deaths, alcohol and/or firearms were involved in 5749 or 63.9%. Higher alcohol and gun law scores were associated with reduced incidence rates and odds of suicides involving either alcohol or firearms (adjusted incidence rate ratios [IRR] 0.72 (95% CI 0.63, 0.83) for alcohol policies, 0.86 (95% CI 0.82, 0.90) for firearm policies). Relationships were similar for suicides involving both alcohol and firearms, and there was an interactive effect, such that states with restrictive policies for both had the lowest rates of suicides involving alcohol or guns.

**Conclusions:**

More restrictive alcohol and firearm policies are associated with lower rates and odds of suicides involving alcohol or firearms, and alcohol and firearms, and may be a promising means by which to reduce suicide.

## Background

Suicide is a leading cause of death in the U.S., and rates have increased by approximately 30% during the past two decades [1]. Alcohol is commonly involved in suicide [2, 3]. The relationship between alcohol consumption, particularly excessive alcohol use and suicide has several mechanisms. First, excessive alcohol use and alcohol use disorder is associated with the development and exacerbation of depression, which is a leading risk factor for suicide [4]. Second, binge drinking, or drinking to the point of impairment, can induce acute dysphoria and inhibit executive functions that may protect against the impulse to commit suicide, even in the absence of known mental illness or ongoing suicidal ideation [1, 5].

Firearms are a common means to commit suicide, and suicides account for approximately two-thirds of all firearm fatalities [6]. From 2001 to 2013, rates of firearm suicide increased in the U.S. [7]. In the U.S., higher rates of firearm ownership and gun availability are associated with increased rates and odds of suicide, particularly among males [8–10].

Across the 50 U.S. states, alcohol and firearm policies vary considerably [11–13]. However, little is known about relationship between alcohol and firearm policies at the state level, relationships between alcohol policies and alcohol-involved suicides, or about the independent or possibly interactive effects of alcohol and firearm policies on state-level rates or individual-level odds of suicides involving alcohol, firearms, or both.

## Methods

The objectives of this lagged cross-sectional study were to assess the relationships between state alcohol and firearm policies and the rates and odds of alcohol and firearm involved suicides, and the interactive effects of such policies.

The methods for this analysis were similar to those described in a previous analysis [14]. Data on suicides came from the Centers for Disease Control and Prevention’s National Violent Death Reporting System (NVDRS) [15–17], which is a census of violent deaths occurring in participating states from sources including death certificates, law enforcement reports, toxicology reports, and coroner/medical examiner records. In 2015, NVDRS captured suicide data from 27 states; among suicide decedents in those states, BAC testing was available on 50.2%. As in our prior research, we excluded the five state-years with < 30% BAC level reporting [14, 18, 19], and in the final analytic sample 62.0% of suicide decedents (8996 persons) in the remaining 22 states had BAC testing (state range 33.3–99.2%).

State alcohol policies were operationalized with Alcohol Policy Scale (APS) scores, which measure the restrictiveness of the alcohol policy “environment” in each state based on 29 individual alcohol policies, also described previously [11]. The scores range from 1 to 100 with higher scores indicating more restrictive policy environments. Higher APS scores have been associated with lower odds of binge drinking among adults and lower odds of drinking and binge drinking among underage youth [11, 20, 21]. For the 22 states represented in the NVDRS data, APS scores ranged from 33.2 to 68.2 (mean 45.7, median 44.3).

Firearm policies were operationalized using scores from the 2014 Gun Law Scorecard from the Giffords Law Center to Prevent Gun Violence (to correspond with the most recent year of available APS scores) [22]. The score assigns point values and sums 34 firearm policies, including those related to private sale background checks, extreme risk protection orders, licensing and registration requirements, restrictions on carrying firearms in public places, permissible firearm types including magazine capacity, and record-keeping with respect to firearms sales or transfers, and local authority to regulate firearms more strictly than the state. Scores could range from 1 to 106 (the maximum theoretical score, representing the most restrictive firearm policy environment). Among the 22 states from NVDRS in 2014, state scores ranged from 8.0 to 85.5 (mean 36.1, median 20.3).

State alcohol and firearm policy scores were from 2014. The two policy measures were correlated among states using Pearson correlation coefficients. We then related alcohol and firearm policy measures in 2014 to state level rates and individual level odds of suicide in 2105 (i.e., using a 1-year lag as in our prior research). Models relating policy scores to state incident rate ratios (IRRs) and adjusted IRRs of firearm and/or alcohol-involved suicide used Poisson regression models. Models of the relationship between policies and the individual-level odds ratios (ORs) and adjusted ORs of alcohol and/or gun involvement in suicides used generalized estimating equations (GEE) to account for county-level clustering of suicides within states. IRRs and ORs were based on an absolute 10 percentage point difference in the policy scores; in each case this represented a meaningful change in state policy that fell well within the ranges of state policies.

State-level covariates included proportions of the population that was male and age ≥ 21, racial and ethnic composition, proportion with a college degree or higher, household income, unemployment rate, police officers per capita, degree of urbanization, and religiosity (proportion Catholic adherents) [23–26]. For adjusted GEE models, we also accounted for individual-level covariates from NVDRS directly including the decedent’s age, sex, race/ethnicity, marital status, and mental health status.

To more fully examine the joint effects of state alcohol and firearm policy scores on suicide, we fit an individual-level multinomial logistic regression model that included an interaction term between the two policy scores, along with state and individual level covariates. The interaction term in this model was significant, and we described the joint effects of the alcohol and gun policy scores by calculating adjusted prevalence rates along with odds ratios and 95% confidence intervals from this model, using mean values for the policy covariates, comparing four state alcohol-firearms policy phenotypes (e.g., states with high-restriction firearm policies, but low-restriction alcohol policies). For these calculations, for both alcohol and firearm policy scores we used our sample’s 25th percentile as a low policy score, and a 10 percentage point increase above that score as constituting a meaningful change towards a higher policy score. The Institutional Review Board at Boston University Medical Campus waived the need for official ethics approval and determined it to be Not Human Subjects Research.

## Results

For the 22 states in 2015, there were a total of 8996 suicide decedents that had BAC test results (Table 1). Of those deaths, 63.9% involved either alcohol or a firearm, including 20.2% that involved alcohol only, 27.6% that involved a firearm only, and 16.1% that involved alcohol and a firearm. Under-age youth aged 20 years or less had the lowest percentage of alcohol-only suicides (10.2%) and the lowest involving alcohol and a firearm (6.4%), but had the highest percentage involving a firearm only (35.7%). Suicides among men, who accounted for almost three-quarters of cases, were more likely to involve firearms-only or firearms and alcohol compared with women (31.4 and 19.1% versus 16.3 and 7.5%, respectively). Persons of white race had the largest percentage of firearm-only suicide, while American Indian/Alaska Natives had the highest percentage of alcohol-only suicide.

**Table 1:** Demographic Characteristics of Suicide Decedents by Alcohol and Firearm Involvement, National Violent Death Reporting System, 2015, United States

	Overall	Neither Firearm- nor Alcohol- Involved	Alcohol- Involved Only	Firearm- Involved Only	Alcohol- and Firearm- Involved	***p***-value
**All Suicide Decedents**	8996	3247 (36.1%)	1817 (20.2%)	2481 (27.6%)	1451 (16.1%)	
**Age**						
< 21	656	313 (47.7%)	67 (10.2%)	234 (35.7%)	42 (6.4%)	<.0001
21 to 29	1359	474 (34.9%)	318 (23.4%)	288 (21.2%)	279 (20.5%)	
30 to 39	1430	482 (33.7%)	365 (25.5%)	299 (20.9%)	284 (19.9%)	
40 to 49	1686	612 (36.3%)	422 (25.0%)	359 (21.3%)	293 (17.4%)	
> =50	3865	1366 (35.3%)	645 (16.7%)	1301 (33.7%)	553 (14.3%)	
**Sex**						
Female	2285	1203 (52.6%)	538 (23.5%)	372 (16.3%)	172 (7.5%)	<.0001
Male	6711	2044 (30.5%)	1279 (19.1%)	2109 (31.4%)	1279 (19.1%)	
**Race/Ethnicity**						
White Non-Hispanic	7391	2534 (34.3%)	1433 (19.4%)	2178 (29.5%)	1246 (16.9%)	<.0001
Black Non-Hispanic	426	182 (42.7%)	92 (21.6%)	74 (17.4%)	78 (18.3%)	
AI/AN Non-Hispanic	166	48 (28.9%)	54 (32.5%)	37 (22.3%)	27 (16.3%)	
Hispanic	616	267 (43.3%)	149 (24.2%)	121 (19.6%)	79 (12.8%)	
Other	397	216 (54.4%)	89 (22.4%)	71 (17.9%)	21 (5.3%)	
**Marital Status**						
No	6068	2293 (37.8%)	1347 (22.2%)	1504 (24.8%)	924 (15.2%)	<.0001
Yes	2786	895 (32.1%)	440 (15.8%)	945 (33.9%)	506 (18.2%)	
**Mental Health Problem**						
No	4510	1344 (29.8%)	851 (18.9%)	1439 (31.9%)	876 (19.4%)	<.0001
Yes	4486	1903 (42.4%)	966 (21.5%)	1042 (23.2%)	575 (12.8%)	

**Fig. 1:**
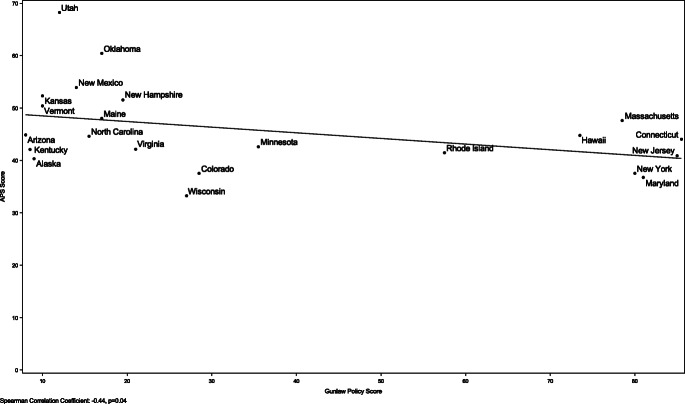
APS Scores and Gun Law Policy Scores for 22 States from NVDRS Dataset, 2014

There was a negative correlation between state-specific policy scores for alcohol and firearms (r = − 0.44, *p* = 0.04, Fig. 1). There was a range of state restrictiveness for both policy measures, but few states had policies that had relatively restrictive policies for both alcohol and firearms.

In state-level multivariable models, we examined associations between the restrictiveness of state alcohol and firearms policy scales (based on a 10 percentage point difference for each scale) and state-level incident rate ratios of suicide (Table 2). In terms of relationships between the alcohol policy scale and suicide rates, in fully adjusted models controlling for state-firearm policies, more restrictive alcohol policies were not associated with alcohol-only suicide rates, but had protective associations for firearm-only suicides (IRR 0.68, 95% confidence interval [CI] 0.55, 0.84), suicides involving alcohol and firearms (IRR 0.48, 95% CI 0.35, 0.66), and suicides involving alcohol or firearms (IRR 0.72, 95% CI 0.63, 0.83).

**Table 2:** Incident rate ratios^a^ and 95% confidence intervals of alcohol- and/or gun-involved suicides using Poisson regression associated with 10 percentage point increase in alcohol policy score or gun policy score category, compared to no alcohol or firearm involvement

	Alcohol Involved IRR (95% CI) (*n* = 1817)	Firearm Involved IRR (95% CI) ((*n* = 2481)	Alcohol- and Firearm- Involved IRR (95% CI) (*n* = 1451)	Alcohol or Firearm InvolvedIRR (95% CI)(*n* = 5479)
**APS Predictor**				
*Model*				
Unadjusted Poisson Model	1.15 (1.08, 1.22)*	1.64 (1.57, 1.71)*	1.46 (1.38, 1.55)*	1.44 (1.40, 1.48)*
Adjusted Poisson Model I (state-level covariates)^b^	1.22 (1.03, 1.44)*	1.48 (1.31, 1.68)*	1.39 (1.18, 1.63)*	1.32 (1.22, 1.44)*
Adjusted Poisson Model II (addition of bac testing rate to model I)	1.11 (0.93, 1.32)	0.89 (0.77, 1.04)	1.00 (0.83, 1.21)	0.98 (0.89, 1.07)
Adjusted Poisson Model III (addition of gun policy score to model II)	1.01 (0.81, 1.28)	0.68 (0.55, 0.84)*	0.48 (0.35, 0.66)*	0.72 (0.63, 0.83)*
**Firearm Policy Predictor**				
*Model*				
Unadjusted Poisson Model	0.97 (0.96, 0.99)*	0.77 (0.75, 0.78)*	0.83 (0.81, 0.84)*	0.85 (0.85, 0.86)*
Adjusted Poisson Model I (state-level covariates)^b^	0.98 (0.93, 1.04)	1.08 (1.01, 1.16)*	0.89 (0.83, 0.94)*	0.98 (0.95, 1.01)
Adjusted Poisson Model II (addition of bac testing rate to model I)	0.95 (0.90, 1.01)	0.95 (0.90, 1.01)	0.86 (0.82, 0.91)*	0.93 (0.90. 0.96)*
Adjusted Poisson Model III (addition of APS score to model II)	0.96 (0.89, 1.03)	0.86 (0.79, 0.93)*	0.74 (0.67, 0.81)*	0.86 (0.82, 0.90)*

a Incident Rate ratio was based on 10 point increase in APS or Gun Law score

b Adjusted model I controls for state proportions of male, age ≥ 21, racial and ethnic composition, college degree or above, household income, unemployment, police rate per capita, degree of urbanization, and religiosity

*Incident Rate Ratios and 95% Confidence Intervals are significant at α = 0.05

For relationships between the firearm policy variable and suicide rates (Table 2), in fully adjusted models controlling for state alcohol policies, more restrictive firearm policies were associated with reduced rates of suicide for firearm-only suicides (IRR 0.86, 95% CI 0.79, 0.93), alcohol and gun-involved suicides IRR (0.74, 95% CI 0.67, 0.81), and alcohol or gun-involved suicides (IRR 0.86, 95% CI 0.82, 0.90). Firearm policies were not associated with alcohol-only suicide rates.

We also assessed individual-level odds of alcohol or firearm involvement among suicide decedents (Table 3), by controlling for individual-level as well as state-level covariates. In general, the odds of alcohol and or firearm involvement were similar in direction, magnitude and statistical significance compared to the incidence rate ratios reported in Table 2. However, there was a significant interaction between the firearm and alcohol policy measures.

**Table 3:** Odds ratios^a^ and 95% CIs of alcohol and/or gun involved suicides associated with 10 percentage point increase in alcohol policy score or gun policy score, compared to no alcohol or firearm involvement

	Alcohol Involved Only OR (95% CI) (*n* = 1817)	Firearm Involved Only OR (95% CI) (*n* = 2481)	Alcohol and Firearm- Involved OR (95% CI) (*n* = 1451)	Alcohol or Firearm- Involved OR (95% CI) (*n* = 5479)
**APS Predictor**				
*Model*				
Unadjusted GEE Model	0.80 (0.72, 0.88)*	1.14 (1.06, 1.23)*	1.02 (0.93, 1.11)	1.00 (0.95, 1.06)
Adjusted GEE Model I^b^ (individual-level covariates)	0.78 (0.70, 0.86)*	1.15 (1.06, 1.23)*	1.02 (0.93, 1.11)	0.99 (0.93, 1.05)
Adjusted GEE Model II^c^ (individual-and state-level covariates)	0.83 (0.62, 1.09)	1.05 (0.86, 1.28)	0.99 (0.71, 1.38)	0.91 (0.74, 1.12)
Adjusted GEE Model III (addition of bac testing rate to model II)	0.96 (0.75, 1.23)	0.94 (0.76, 1.16)	1.06 (0.76, 1.50)	0.97 (0.78, 1.20)
Adjusted GEE Model IV (addition of gun policy score to model III)	0.84 (0.60, 1.17)	0.62 (0.47, 0.81)*	0.46 (0.30, 0.71)*	0.66 (0.51, 0.85)*
**Firearm Policy Predictor**				
*Model*				
Unadjusted GEE Model	1.06 (1.02, 1.10)*	0.83 (0.80, 0.85)*	0.89 (0.86, 0.93)*	0.93 (0.90, 0.95)*
Adjusted GEE Model I (individual-level covariates)	1.07 (1.03, 1.11)*	0.81 (0.79, 0.84)*	0.89 (0.85, 0.93)*	0.93 (0.90, 0.96)*
Adjusted GEE Model II (individual-and state-level covariates)	0.93 (0.82, 1.05)	0.92 (0.86, 0.99)*	0.81 (0.70, 0.94)*	0.90 (0.80, 0.99)*
Adjusted GEE Model III (addition of bac testing rate to model II)	0.96 (0.84, 1.10)	0.90 (0.84, 0.96)*	0.82 (0.71, 0.95)*	0.90 (0.81, 1.01)
Adjusted GEE Model IV (addition of APS to model III)	0.92 (0.77, 1.10)	0.79 (0.72, 0.87)*	0.68 (0.57, 0.82)*	0.82 (0.71, 0.94)*

Referent group is neither gun nor alcohol involved

a Odds ratio was based on 10 point increase in APS or Gun Law score

b Adjusted GEE model I controls for victim’s age, sex, race/ethnicity, marital status, and mental health status

c Adjusted GEE model II additionally controls for state proportions of male, age ≥ 21, racial and ethnic composition, college degree or above, household income, unemployment, police rate per capita, degree of urbanization, and religiosity

*Odds Ratios and 95% Confidence Intervals are significant at α = 0.05

A multinomial logistic regression model that included the interaction term between the alcohol and gun state policy scores, controlling for state and individual level covariates, found a significant interaction between the two policy scores. From this model, we calculated adjusted prevalence and odds ratios comparing four state alcohol-firearms policy phenotypes (e.g., high-restriction firearm policies, low-restriction alcohol policies) (Table 4). States with restrictive policies for both alcohol and firearms had the highest percent of suicides involving *neither* alcohol nor guns, and the lowest percent involving *both* alcohol and guns. More restrictive firearm policies were associated with reduced gun-involved suicides (with or without alcohol involvement), and a further decrease in odds of gun involvement (with or without alcohol) was observed with both more restrictive alcohol and firearm policies than with more restrictive firearm policies alone. Similarly, decreases in odds of alcohol involvement were observed with both more restrictive firearm and alcohol policies than with more restrictive alcohol policies alone.

**Table 4:** Adjusted prevalence distribution and adjusted odds of alcohol- and gun-involved suicides, by state alcohol and gun policy score phenotypes from multinomial logistic regression model including interaction between alcohol and gun policy scores

Alcohol Policy Score^b^	Gun Policy Score^b^	Neither Alcohol nor Firearm Suicides	Alcohol Only Suicides	Firearm Only Suicides	Alcohol and Firearm Suicides
		adjusted % AOR (95% CI)	adjusted % AOR^a^ (95% CI)	adjusted % AOR^a^ (95% CI)	adjusted % AOR^a^ (95% CI)
Low	Low	20.6% Ref	10.1% Ref	51.6% Ref	17.6% Ref
High	Low	30.5% Ref	17.2% 1.21 (0.85, 1.72)	33.3% 0.42 (0.27, 0.66)	19.1% 0.79 (0.55, 1.13)
Low	High	25.4% Ref	11.9% 0.95 (0.80, 1.13)	46.6% 0.73 (0.65, 0.82)	16.1% 0.74 (0.63, 0.87)
High	High	37.4% Ref	15.5% 0.89 (0.55, 1.43)	35.4% 0.37 (0.23, 0.59)	11.7% 0.40 (0.24, 0.65)

^a^ Adjusted odds ratios compare the odds of the suicide category (columns in the table) for states with a APS – gun score combination vs. states with low scores on both the APS and gun score

^b^ Low policy scores are at the sample 25th percentile (40.9 for the APS score, 12 for the gun policy score), high policy scores are 10 points higher than the low score. Lower scores indicate less restrictive policy environments, whereas higher scores indicate more restrictive policy environments

## Discussion

This study found that more restrictive alcohol and firearm policies were associated with lower rates and odds of alcohol and firearms suicides, and found that there was a significant interaction between alcohol and firearms policies. These protective relationships were particularly striking for suicides involving both alcohol and firearms.

Because this was a cross-sectional analysis, this should be considered a hypothesis-generating study that cannot prove a causal association between alcohol or firearm policies and suicide. Furthermore, using a lag between policies and suicide outcomes does not eliminate reverse causation as a possible explanation for the results. However, previous studies incorporating multiple years of data have found that more restrictive alcohol control policies protect against suicide, alcohol-involved suicide, and firearm injury rates [27, 28]. In addition, less restrictive firearm policies were associated with increased risk of suicide and homicide [13, 29, 30]. Our previous research finds that more restrictive alcohol policies reduce the odds of alcohol involvement among homicide victims [18]. Therefore, in the context of the broader literature, the results are plausible.

In addition to the cross-sectional study design, results are subject to other limitations. NVDRS data were only available for 22 states in 2015, and so our findings are not nationally representative. We could only assess 1 year of data because the availability of the alcohol and policy variables only overlapped in 2014. It is unclear why protective associations between alcohol policies and alcohol-involved suicides were somewhat lower than we found in a previous analysis [31]. However, the previous analysis involved more years of data (from 2003 to 2012) but among only the 13 states covered by NVDRS during that time period. Relatively lower rates of testing for alcohol may result in selective testing in which those suspected of using alcohol are more likely to be tested; to address this potential source of bias we included the BAC testing rate as a state-level covariate in adjusted models. Finally, there could be confounding factors for which could have influenced the associations reported in this study.

Although models examining individual-level odds of alcohol or firearm involvement in suicide have the advantage of accounting for individual-level confounders, and also control for other state level factors that may influence rates, our findings for relationships between rates versus odds of policy-suicide outcomes were consistent, which adds to the robustness of the findings.

There was a strong protective interaction between alcohol and firearm policy variables, particularly for suicides involving alcohol. Previous research suggest young and middle-aged persons who commit suicide with firearms are more likely to use alcohol than those who commit suicide by poisoning or other means [2, 32]. In addition, those with both acute and chronic excessive drinking are more likely to own firearms, and to be victims of firearm suicide [33, 34]. Therefore, because there is evidence that more restrictive alcohol policies are associated with lower rates of excessive drinking [11, 35], and that more restrictive firearm policies are associated with reduced firearms ownership, these results seem plausible and suggest that increasing the restrictiveness of both alcohol and firearm policies, rather than either in isolation, are promising for reducing suicide. These findings also suggest that laws restricting firearms ownership among high-risk individuals (so-called ‘may issue’ laws), including those who drink excessively or have experienced alcohol-related criminal offenses, may reduce firearm suicides [29].

## Conclusions

Stronger alcohol and firearm policies are a promising means to prevent a leading and increasing cause of death in the U.S. The findings further suggest that strengthening both policy areas may have a synergistic impact on reducing suicides involving either alcohol, firearms, or both. In future research, longitudinal studies using multiple years of policy and suicide data would strengthen causal inference.

## Data Availability

Data on suicide decedents was obtained from the National Violent Death Reporting System’s Restricted Access Database, which is available to researchers meeting certain criteria. The study team completed a research proposal and obtained permission to access the restricted database. Further details can be found here: https://www.cdc.gov/violenceprevention/datasources/nvdrs/datapublications.html. The Alcohol Policy Scale is available from the corresponding author on reasonable request.

## Abbreviations

BAC: Blood alcohol concentration
IRR: Incidence rate ratio
NVDRS: National Violent Death Reporting System
APS: Alcohol Policy Scale
BRFSS: Behavioral Risk Factor Surveillance System
OR: Odds ratio
GEE: Generalized estimating equations
